# MEG-Derived Symptom-Sensitive Biomarkers with Long-Term Test-Retest Reliability

**DOI:** 10.3390/diagnostics12010084

**Published:** 2021-12-30

**Authors:** Don Krieger, Paul Shepard, Ryan Soose, Ava Puccio, Sue Beers, Walter Schneider, Anthony P. Kontos, Michael W. Collins, David O. Okonkwo

**Affiliations:** 1 Department of Neurological Surgery, University of Pittsburgh, Pittsburgh, PA 15217, USA; puccam@upmc.edu (A.P.); okonkwodo@upmc.edu (D.O.O.); 2 Department of Physics and Astronomy, University of Pittsburgh, Pittsburgh, PA 15217, USA; shepard@pitt.edu; 3 Department of Otolaryngology, University of Pittsburgh, Pittsburgh, PA 15217, USA; soosrj@upmc.edu; 4 Department of Psychiatry, University of Pittsburgh, Pittsburgh, PA 15217, USA; BeersSR@upmc.edu; 5 Department of Psychology, University of Pittsburgh, Pittsburgh, PA 15217, USA; wws@pitt.edu; 6 Department of Sports Medicine, University of Pittsburgh, Pittsburgh, PA 15217, USA; kontosap@upmc.edu (A.P.K.); collinsmw@upmc.edu (M.W.C.)

**Keywords:** CamCAN, TEAM-TBI, post-concussion syndrome, test-retest reliability, insomnia, depression, anxiety, somatization, pain, sleep disorder, ocular, vestibular

## Abstract

Neuroelectric measures derived from human magnetoencephalographic (MEG) recordings hold promise as aides to diagnosis and treatment monitoring and targeting for chronic sequelae of traumatic brain injury (TBI). This study tests novel MEG-derived regional brain measures of tonic neuroelectric activation for long-term test-retest reliability and sensitivity to symptoms. Resting state MEG recordings were obtained from a normative cohort (CamCAN, baseline: *n* = 613; *mean* 16-month follow-up: *n* = 245) and a chronic symptomatic TBI cohort (TEAM-TBI, baseline: *n* = 62; *mean* 6-month follow-up: *n* = 40). The MEG-derived neuroelectric measures were corrected for the empty-room contribution using a random forest classifier. The *mean* 16-month correlation between baseline and 16-month follow-up CamCAN measures was 0.67; test-retest reliability was markedly improved in this study compared with previous work. The TEAM-TBI cohort was screened for depression, somatization, and anxiety with the Brief Symptom Inventory and for insomnia with the Insomnia Severity Index and was assessed via adjudication for six clinical syndromes: chronic pain, psychological health, and oculomotor, vestibular, cognitive, and sleep dysfunction. Linear classifiers constructed from the 136 regional measures from each TEAM-TBI cohort member distinguished those with and without each symptom, *p* < 0.0003 for each, i.e., the tonic regional neuroelectric measures of activation are sensitive to the presence/absence of these symptoms and clinical syndromes. The novel regional MEG-derived neuroelectric measures obtained and tested in this study demonstrate the necessary and sufficient properties to be clinically useful, i.e., good test-retest reliability, sensitivity to symptoms in each individual, and obtainable using automatic processing without human judgement or intervention.

## 1. Introduction

Traumatic brain injury (TBI) is a common cause of disability and death. Localized neuroelectric correlates of persistent functional sequelae after TBI would provide significant clinical value for diagnosis, targeted therapy, disease monitoring.

For more than a century It has been the expectation that neuronal electric activity is the key to understanding the brain function. Human behavior is thought to depend on cooperative activity of large neural populations. Today, clinical neurophysiologists routinely measure single neurons to aide implantation of therapeutic devices deep in the brain [1]. Epileptologists map intractable seizures with implanted “stereo EEG” electrodes [2]. Indwelling electrical recordings have resolution of a few millimeters at best. The spatial resolution for non-invasive scalp recordings is considerably poorer. 

Magnetoencephalography (MEG) is also noninvasive and has important advantages over scalp and even direct electrical recordings. High fidelity measurements of the magnetic fields due to neuroelectric currents are routinely obtained at the MEG sensor array Since magnetic fields do not interact with brain tissue [3,4,5,6] as electric fields do, neuroelectric currents are more readily localized via MEG than EEG. 

The common presumption is that cortical population post-synaptic currents are the source of MEG recordings. [4,5,6] Most reported work therefore limits the volumes in which neural currents may be localized to the cerebral cortex. although there are occasional reports in which neuroelectric dipoles are localized to the white matter, e.g., [7]. Additional remarks are found in the **Discussion** section.

The referee consensus solver utilizes a method which extracts profuse validated and localized (1 mm resolution) neuroelectric current waveforms (*p* < 10^−12^ for each), from cortical, subcortical and white matter volumes [8,9,10]. Recordings from healthy volunteers (CamCAN, *n* = 621) were processed to generate a normative database, including metrics of test-retest reliability (*n =* 253) across MEG studies repeated 16 months apart. Good test-retest reliability is a primary requirement for both scientific and diagnostic usefulness. 

Recordings were processed from a cohort of chronic TBI subjects with persistent symptoms (Team-TBI, *n =* 64) to identify patterns of brain activity which distinguish between those with and without specific TBI sequelae. A second critical requirement for clinical usefulness is sensitivity to clinical symptoms.

Validation of clinically useful neuroelectric brain measures is the primary objective of our effort. 136 brain regions were identified for each subject from both cohorts. Each individual’s regional measures were transformed to *z-scores* using the *means* and *standard deviations* from the CamCAN cohort baseline results. Test-retest reliability was assessed using each CamCAN cohort member’s baseline and follow-up *z-scores*. Sensitivity to symptoms was assessed using each TEAM-TBI cohort member’s baseline and follow-up *z-scores*. 

The data processing pipeline functions without human judgement or intervention and is capable of fully processing each new recording within 24 h [8]. This is consistent with the translational objective of the effort.

High-fidelity waveforms are localized to 1 mm^3^ [3,4] for dipole electric currents with 80 ms duration. The normalized count of these instances within each standardized region is the measure used in the present study, i.e., each measure is the total value over a brain region automatically identified from one subject’s scan. The fact that each standardized region is automatically identifiable (freesurfer 5.3, [11,12]) for each scan enables generating standardized measures, i.e., norms, for each region across a large normative cohort. The values for those regions in any individual may then be compared with the norms to assess the normality of the individual’s regional measures. 

Note that the accuracy of each region’s volume and freesurfer’s parcellation limit the accuracy of each measure’s localization. The regional volumes range from less than 1.0 cm^3^, e.g., left or right nucleus accumbens, to 30–70 cm^3^, e.g., left or right cerebellar cortex.

The results reported here enable rejection of the following null hypotheses. (a) Individuals with and without symptoms are indistinguishable. (b) The cohort membership of each individual (TEAM-TBI or CamCAN) cannot be determined. (c) Regional measures from an individual do not reliably repeat.

Joint rejection of hypotheses (a) and (c) supports the potential for these measures as clinically useful in the diagnosis and treatment of insomnia, depression, anxiety, somatization, chronic pain, psychological health, and vestibular, oculomotor, sleep, and cognitive dysfunction, common sequelae of TBI. Rejection of hypothesis (b) suggests the possibility that these measures may be useful as biomarkers for TBI. It is symptoms rather than etiology which is the emphasis in the study design. It is hoped that this shift in focus will produce insights which are useful in diagnosis and treatment of those symptoms, regardless of etiology. Repeated MEG recordings during drug or other treatment modalities may provide objective and useful information in assessing treatment efficacy and in making adjustments to the treatment.

The current study is a natural follow-on to our most recent report [10] with two notable additions. (1) The MEG-derived measures used there were corrected for empty-room contributions using a weaker method. Here we used a random forest classifier to identify individual electric currents. (2) The symptom measures used there were insomnia, depression, anxiety, and somatization as assessed using self-report symptom surveys. Here we use those symptoms and six additional clinical syndromes assessed via adjudication. 

## 2. Materials and Methods

Magnetoencephalographic (MEG) recordings were processed from each subject of two cohorts: (1) the normative CamCAN cohort, *n* = 621 at baseline, ages 18–87 [13,14], *n* = 259 at follow-up, and (2) the chronically symptomatic concussed TEAM-TBI cohort, *n* = 63 at baseline, ages 21–60, *n* = 40 at follow-up. The MEG recordings were coregistered with a high-resolution T1-weighted MRI scan obtained at baseline.

Validation of clinically useful neuroelectric brain measures is the primary objective of our effort. Each individual’s regional measures were transformed to *z-scores* using the *means* and *standard deviations* from the CamCAN cohort baseline results. Test-retest reliability was assessed using each CamCAN cohort member’s baseline and follow-up *z-scores* Sensitivity to symptoms was assessed using each TEAM-TBI cohort member’s baseline and follow-up *z-scores*. The data processing pipeline functions without human judgement or intervention and is capable of fully processing each new recording within 24 h [8].

The raw MEG data from each subject was initially transformed to a collection of probabilistically validated neuroelectric currents. Each current is 80 ms in duration and is localized with 1 mm (mm) and 1 millisecond (ms) resolution. The total current count per subject per minute yielded by this primary processing step is typically in excess of 500,000.

The current counts were normalized to produce measures of tonic activity for each of 171 standard regions of interest (ROIs): 17 subcortical regions, 68 cortical regions, 68 adjacent white matter regions, and 18 deep white matter tracts. Each regional measure is a count of all the neuroelectric currents localized within the region over the several-minute recording time. The regional current count is high; hence the available statistical power is high.

### 2.1. CamCAN Dataset

The Cambridge Centre for Ageing and Neuroscience (CamCAN) Stage 2 cohort study is a large cross-sectional adult lifespan study (ages 18–87) of the neural underpinnings of successful cognitive ageing [13,14]. The work reported here utilized the majority subset (*n* = 621) of the cohort for whom high resolution (1 mm) anatomic T1-weighted MR imaging and MEG recordings were available. Of these, 253 follow-up resting recordings were obtained (Stage 3 longitudinal study) with a *mean* interval of 16 months between MEG studies. Diffusion-weighted imaging (DWI) was obtained for 589 of the baseline subjects, 240 of whom returned for follow-up. 

MR imaging was obtained on all subjects at a single site using a 3T Siemens TIM Trio scanner, Siemens Healthcare, Camberley, UK, with 32-channel head coil. T1 scans were obtained using the MPRAGE sequence. The field of view for these scans was 256 × 240 × 192 at 1 mm resolution. DWI scans were acquired (*n* = 589) with a twice-refocused spin-echo sequence, with 30 diffusion gradient directions for each of two b-values: 1000 and 2000 s/mm^2^, plus three images acquired with a b-value of 0. Other parameters are: TR = 9100 ms, TE = 104 ms, voxel size = 2.0 mm, FOV = 96 mm × 96, 66 axial slices [13].

MEG recordings with continuous head position measures were collected at a single site using a 306-channel VectorView MEG system (Elekta Neuromag, Helsinki, Finland). The data were sampled at 1 KHz with anti-aliasing low-pass filter at 330 Hz and high-pass filter at 0.03 Hz. Subjects were seated upright for all recordings.

Eyes closed resting recordings for 560 s were collected at baseline [13]. Eyes open recordings for 560 s during performance of a sensorimotor task were collected in the same sitting (*n* = 619). Baseline resting and sensorimotor task recordings were compared to assess short-term test-retest reliability.

For the sensorimotor task, subjects detected visual and auditory stimuli and responded to detection of each with a button press with the right index finger. The stimuli were two circular checkerboards presented simultaneously to the left and right of a central fixation cross, 34 ms duration, and a binaural tone of 300 ms duration. The tone was at 300, 600, or 1200 Hz in equal numbers with the order randomized. 121 trials were presented with simultaneous visual and auditory stimulation. Eight trials were randomly intermixed in which one stimulus was presented at a time, four visual and four auditory. This was done to discourage dependence on one stimulus modality only. The average inter-trial interval was approximately 4.3 s.

At follow-up, 320 s were recorded continuously with eyes closed resting, *n* = 253 [14] and 60 s of empty-room recordings were also obtained.

### 2.2. TEAM-TBI Dataset

The chronic TBI subject dataset was derived from the Targeted Evaluation, Action and Monitoring of Traumatic Brain Injury (TEAM-TBI) study, a personalized medicine research program for subjects with chronic TBI sequelae at the University of Pittsburgh (clinicaltrials.gov: NCT02657135). All TEAM-TBI subjects gave their informed consent for inclusion before the participated in the study. The study was conducted in accordance with the Declaration of Helsinki, and the protocol was approved the Institutional Review Board of The University of Pittsburgh (PRO13070121).

Inclusion criteria were ages 18–60 with a history of one or more TBIs more than six months prior, with high chronic symptom load [15] as assessed with post-concussion symptom severity (PCSS) scale. 61 of the 63 subjects with MEG recordings had sustained “mild” TBIs. TEAM-TBI subjects underwent a 4-day comprehensive clinical assessment, including advanced neuroimaging, followed by multi-disciplinary adjudication of clinical syndromes. TEAM-TBI subjects then completed 6 months of supervised, targeted therapy. Subjects returned to Pittsburgh for a follow-up evaluation (*mean* interval = 6.4 months) to document impact of treatment on identified clinical disorders.

MR imaging was obtained on all subjects at a single site using a 3T Siemens TIM Trio scanner, Siemens Medical Solutions USA, Malvern, PA, USA with 32-channel head coil. T1 scans were obtained using the MPRAGE sequence. The field of view for these scans was 256 × 256 ×176 at 1 mm resolution. DWI scans were acquired (*n* = 64) with a twice-refocused spin-echo sequence, with 64 diffusion gradient directions at b-values of 1000 and 3000 s/mm^2^, and 128 directions at b-values of 5000, and 7000. Additional parameter for the four b-values were: TR = 3700, 3700, 4100, 4500 ms, TE = 92,125,147,164 ms. voxel size = 2.4 mm, FOV = 230.4 mm, 63 axial slices.

MEG recordings were collected at a single site using a 306-channel VectorView MEG system, Elekta Neuromag, Helsinki. The data were sampled at 1 KHz with anti-aliasing low-pass filter at 330 Hz and high-pass filter at 0.03 Hz. Continuous head position measures were enabled throughout the recordings. All recordings were obtained with the subject sitting up.

At baseline four 200-s resting recordings were obtained with eyes open and fixated with the room darkened (*n* = 63). Four to eight recordings were obtained totaling 1500 s with the lights on during performance of a visual semantic decision task [8]. The protocol was the same at follow-up (*n* = 40).

All baseline resting MEG recordings were used (*n* = 63) for subjects whose high resolution (1 mm) anatomic T1 and MEG recordings were available. Of these, 40 follow-up resting recordings were obtained, *mean* interval = 6.4 months. DWI was obtained for 63 of the 64 baseline recordings and 39 of the 40 follow-up recordings. At both baseline and follow-up sessions 300 s of empty-room recordings were obtained.

### 2.3. MRI Processing

Each high resolution T1 scan was processed with Freesurfer, version 5.3, using its default Desikan-Killiany atlas parcellation [11,12]. Freesurfer is a segmentation package which automatically and reliably identifies brain regions. The 3-dimensional coordinates of the extent of the brain volume and 153 standardized regions of interest (ROI’s) were identified, 68 cortical regions, 68 adjacent white matter rims of tissue with thickness ≤ 5.0 mm, and 17 subcortical regions.

### 2.4. MEG Processing

The MEG channels were each filtered using MNE tools with high and low pass at 10 and 250 Hz, 5 Hz roll-off [16]. 250 Hz was used for the low pass to thoroughly remove the continuous head positioning signals present in the raw MEG at 293, 307, 314, and 321 Hz. Previous work has shown that the higher the low-pass frequency, the greater is the yield of the solver [8,9]. Note that the 10 Hz high pass filtering effectively demeans each channel and removes much of the low frequency content most commonly studied. 10 Hz was used for the high pass because (a) the solver yield significantly increases with the low frequencies removed and (b) the solver was set to search one 80 ms data segment at a time. Data lengths greater than this produce reduced solver yield [8,9], presumably because current dipole orientation rarely remains stable for that long. This short data length provides very low sensitivity to frequencies below 12 Hz. However, the solver was stepped through the data in 40 ms increments; hence a bolus of identified current dipoles was identified at 25 Hz. Analysis of the time course of those boluses can provide analysis of low frequencies. That work is outside the scope of the present study.

For each 1.24 s data segment, mains noise was removed from the CamCAN data at 50, 100, 150, 200, and 250 Hz using polynomial synchronous noise removal [17]. Mains noise was removed at 60, 120, 180, and 240 Hz from the TEAM-TBI data. No other preprocessing was applied, and no data segments were excluded by manual artifact identification.

The subject’s head position within the MEG scanner was manually coregistered to the TI scan using Elekta’s Mrilab visualization tool. The coordinates of the center point of a sphere most nearly approximating the brain were identified. These are the only operations in the data processing pipeline for which human judgement was applied. All other operations were fully automated.

Continuous head positioning measures were extracted using Elekta’s MaxFilter tool [18]. The coregistration of the MEG sensor array with the location of the subject’s head and brain was corrected once per second using the continuous head positioning information. This correction was applied to the forward solution used by the solver. The referee consensus solver is described in detail elsewhere [8,9,19].

The forward solution is the mathematical relationship between a putative electric current within the brain and the resultant magnetic field measurements at the sensor array. The solution we used models the brain as a uniformly conducting sphere [3]. Currents within 30 mm of the center of the sphere are nearly undetectable and the mathematical formulation for the forward solution is poorly behaved for this volume; hence it was excluded from the search. The excluded volume typically includes the posterior thalamus, the posterior commissure, and much of the midbrain. The solver’s search volume was delimited using the automated brain segmentation provided by Freesurfer with the 30 mm sphere at the center excluded.

The solver was deployed on The Open Science Grid, an international distributed supercomputing partnership for data-intensive research [20,21]. The work described here utilized more than 70,000,000 processor-hours on the OSG. The solver is detailed in [8,9,10].

When applied to continuous MEG recordings, the solver typically identifies and validates more than 400 neuroelectric currents within the brain per 40-ms step through the data stream, *p* < 10^−12^ for each, *p* < 10^−4^ for each when conservatively corrected for multiple comparisons (Bonferroni). That is more than 600,000 currents per minute of recorded MEG data identified with millimeter and millisecond resolution. Note that data segments contaminated by movement or other artifacts were not manually identified for removal. Instead, artifact rejection relied upon the referee consensus solver’s inherent failure to validate neuroelectric currents when presented with noisy data [10,19].

The validated currents within each of the 171 automatically identified brain regions were counted over the duration of the recording. Each count was normalized to current density, ρ_roi_:

ρ_roi_ = (count_region_/count_total_) ÷ (vol_region_/vol_total_) (1)

The purpose of this normalization is to enable comparisons of a region within or between individuals, during different states, at different times, or comparisons of one region with another. The normalization is defined so that ρ_region_ = 1.0 for all regions if the neuroelectric currents are uniformly distributed throughout the brain. In that isotropic case, (a) the regional count fraction is always equal to the regional volume fraction and (b) no difference is found for any comparison. Dividing the counts for a region by the total count normalizes ρ for variations due to both data quality and record length. The normalization for data quality is important since the yield of the solver changes from moment to moment as data quality waxes and wanes [8,19].

### 2.5. Normative Measures

Normative values for regional measures of static neuroelectric activity were established. To accomplish this, the *mean_ρ_* and *standard deviation_ρ_* for each regional current density (ρ) were obtained from the CamCAN recordings. Ρ for any region for any individual may then be compared with the norm for that region by converting it to a *z-score* with corresponding *p*-value under the assumption that the current densities for the normative population are normally distributed.

*z-score_ρ_* = (ρ – *mean*_ρ_) ÷ (*sd*_ρ_) (2)

The tables of CamCAN *means* and *standard deviations* are presented in Appendix A Table A1. They constitute an atlas which may be used to transform the current densities from any individual to *z-scores* and then assess the normality of deep white matter tonic neuroelectric traffic and cortical/subcortical tonic neuroelectric activity.

Note that transformation of the density measures to *z-scores* nominally equalizes the variances of the norms for all of the regions. This insures that for a composite measure, e.g., a linear classifier composed of the 18-tract *z-scores*, the impact of each of the 18 densities is approximately equal.

### 2.6. Empty Room Correction

**Figure 1:**
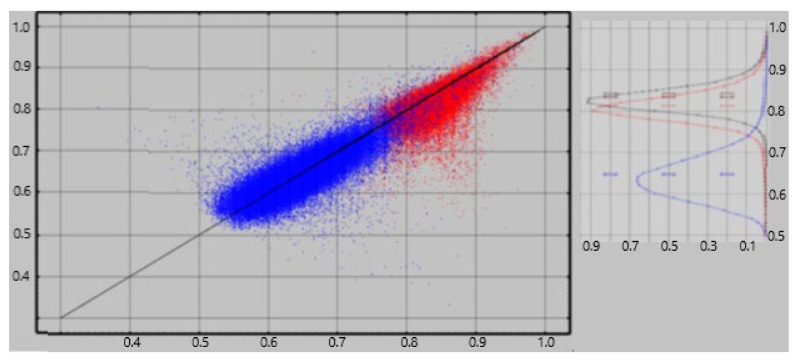
Random forest classification accuracy. Results for all CamCAN subjects (*n* = 613) x all regions (*n* = 168) are shown. **Red:** Each dot in the left panel represents the classification accuracy for empty-room (*x*-axis) vs resting (*y*-axis) data. All of the 80-ms currents were used for a single region for a single subject. See the text for detail. **Blue:** Each dot represents classification accuracy for task (*x*-axis) vs resting (*y*-axis) data. The right panel shows histograms of the resting classification accuracies for resting vs. empty-room (red) and resting vs. task (blue).

An ideal method for extracting neuroelectric measures from MEG recordings would consistently yield a value of zero from empty room recordings. The referee consensus solver falls short of this ideal—there is a significant contribution of falsely validated currents, i.e., “dark count”. The normalized current densities extracted from empty room recordings consistently demonstrate significant correlations to the densities extracted from human resting recordings obtained the same day ([10], Figure 1). We previously used an approximate correction to each ρ_region._ That correction used the correlations as estimates of the competitive detection advantages of true vs. false neuroelectric currents, viz.

ρ_region-corrected_ = ρ_region_ − (*corr*_region_ × ρ_region-empty_) (3)

Here ρ_region-empty_ is the result of the ρ_region_ calculation applied to the empty-room data and *corr*_region_ is the correlation across the CamCAN subjects between ρ_region_ and ρ_region-empty_, i.e., between the regional activity measured with the subject present and absent in the scanner.

For cortical and adjacent white matter regions, we applied a more accurate correction using a random forest classifier [22] to decide on inclusion/exclusion of each identified current one at a time. A separate classifier was computed for each of 136 regions for each subject. The size of the training subset was limited to 5000 currents from each of the two current populations being classified, viz. resting vs. empty room and resting vs. task. This training set limitation reduces the computation time and memory requirements of the calculation while providing training set size which is ample to produce reliable results. In addition, the number of currents from the larger population was limited to 110% of the number of currents in the smaller population if the smaller population’s size was less than 5000. This constraint to approximately equal sizes for the two classes eliminates a key failure point for random forest classification [22]. The resultant classifier was then applied to the total populations, i.e., training + test, to obtain the percentage accuracy for each. The results are shown in Figure 1. The figure shows classification accuracies of 75–95% for rest vs. empty room “currents” compared with 53–75% for rest vs task. Hence empty room “currents” differ more reliably from resting currents than do resting and task currents from each other.

Each current was characterized using four features. The features were defined from the following quantities. Each current has a location, I_xyz_, and $\vec{I_{\mathrm{xyz}}}$, a direction vector in the plane which is normal to the head-approximating sphere at I_xyz_. ctx|wm_xyz_ is the location of the point on the interface between gray and white matter nearest to the current location, I_xyz_. pia_xyz_ is the location of the point on the pial nearest to ctx|wm_xyz_. $\vec{\mathrm{ctx}|{\mathrm{wm}}_{\mathrm{xyz}}}$ is the vector normal to the gray/white interface nearest location I_xyz_. $\vec{r_{\mathrm{xyz}}}$ is the radial vector originating at the origin of the head-approximating sphere and passing through location I_xyz_.

Four local features were identified for each identified electric current, I_xyz_.

1. cos($\vec{I_{\mathrm{xyz}}}$, $\vec{\mathrm{ctx}|{\mathrm{wm}}_{\mathrm{xyz}}}$)
2. cos($\vec{r_{\mathrm{xyz}}}$, $\vec{\mathrm{ctx}|{\mathrm{wm}}_{\mathrm{xyz}}}$)
3. distance(I_xyz_, ctx|wm_xyz_)
4. distance(pia_xyz_, ctx|wm_xyz_), i.e., cortical thickness at I_xyz_

**Figure 2:**
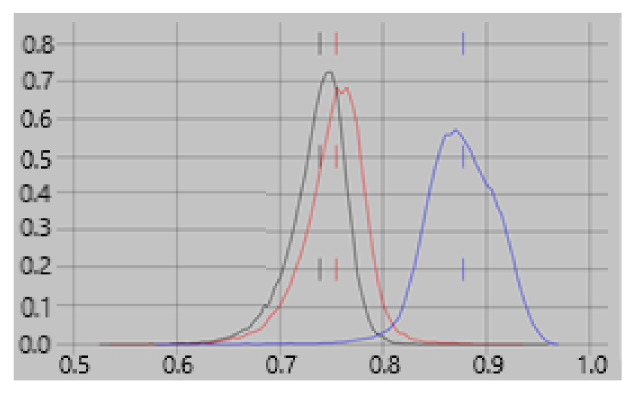
Feature contribution to classification accuracy. Histograms of the contribution of feature #1 to classification accuracy are shown for empty-room vs. rest (red), empty-room vs. task (black), and rest vs. task (blue). See text for details. Of the four features used in the classifications, #1 depends most heavily on the direction of current flow. It is expected to make a greater contribution to classifiers for one brain state vs. another than for a brain state vs. empty-room results. The figure confirms this expectation.

All four features depend on both the location of the current, I_xyz_, and the geometry of the brain within a few mm of I_xyz_. Feature 1 also depends on the direction in which the current flows. Since Feature 1 is more dependent on a neuroelectric current properties than the other features, we expect it to have a greater contribution to classifiers for one brain state vs another, e.g., rest vs task, than for classifying empty room vs brain recordings. This expectation is confirmed in Figure 2 which shows that the contribution of feature #1 to the rest vs task classifier is consistently greater than for empty room vs rest or task.

### 2.7. Classification

Regional measures of neuroelectric activity for 68 cortical regions and 68 adjacent white matter regions were combined into classifiers using stepwise linear classification [17,18,19]. This is an automated computer algorithm which performs discriminant analysis between two groups by computing a linear classification function in a stepwise manner. The groupings for classification were determined (a) by symptom survey scores and (b) by consensus adjudicated opinions to test for sensitivity of the measures to symptoms and by cohort membership to test for differences between the cohorts. These are detailed in the **Results** section.

## 3. Results

Validation of clinically useful neuroelectric brain measures is the primary objective of our effort. Each individual’s regional measures were transformed to *z-scores* using the *means* and *standard deviations* from the CamCAN cohort baseline results. Test-retest reliability was assessed using each CamCAN cohort member’s baseline and follow-up *z-scores*. Sensitivity to symptoms was assessed using each TEAM-TBI cohort member’s baseline and follow-up *z-scores*. 

Regional measures of neuroelectric activity for 68 cortical and 68 adjacent white matter regions were extracted from the MEG recordings for each study participant. For the normative CamCAN cohort, the *mean* and *standard deviation* baseline values for each region are shown in Appendix A Table A1. These values were used to transform all regional measures to *z-scores*. Appendix A Table A2 shows the *correlations* and *differences* for baseline vs. follow-up measures. These were used to assess test-retest reliability. The presence/absence of relationships between these neuroelectric measures and measures of potential clinical relevance was tested.

This effort has produced several types of results. Significant relationships were found between measures of tonic regional neuroelectric activity and (1) screening measures of insomnia, depression, anxiety, and somatization, (2) adjudicated assessments of six clinical syndromes: chronic pain, psychological health, and cognitive, ocular, sleep, and vestibular dysfunction, and (3) subjects in the CamCAN control cohort vs. the TEAM-TBI chronically symptomatic group with history of concussion. These bear directly on the potential usefulness of the measures as diagnostics and as probes for scientific questions. 

In addition (4) both short-term (1-h) and long-term (16-month) baseline vs. follow-up test-retest reliability results are reported. This too bears directly on potential clinical utility. Note that all regional neuroelectric activity measures were reduced to *z-scores*; the means and standard deviations of the baseline CamCAN recordings (*n* = 613) were used for the z-score transformation.

### 3.1. Self-Reported Symptoms and Adjudicated Clinical Syndromes

MEG-derived neuroelectric measures were obtained from 63 TEAM-TBI subjects at baseline and from the 40 who returned for follow-up. Symptom surveys for insomnia and three symptoms of psychological distress were obtained from all but one of the baseline subjects. Standard inventories were for insomnia (Insomnia Severity Index, ISI, [23,24]) and somatization, depression and anxiety (Brief Symptom Inventory, BSI, [25,26]. Cut-offs of 15 (ISI) and 63 (BSI) were used to divide both the baseline and follow-up TEAM-TBI recordings into clinically negative or positive groups. In addition, adjudicated binary assessments of six clinical syndromes were obtained: chronic pain, psychological health, and cognitive, ocular, sleep, and vestibular dysfunction. The matrix of coincidence rates between the symptoms and clinical syndromes is shown in Table 1. Note that the acceptance criteria for the TEAM-TBI study included “high symptom burden.”

Regional measures of neuroelectric activity for 68 cortical regions and 68 adjacent white matter regions were combined into classifiers using stepwise linear classification [27,28,29]. Classification accuracies with *p*-values are shown in Table 2. 

The *p*-values were computed as follows. Consider line 1 of the table: 45 of 53 TEAM-TBI subjects who screened negative for insomnia were classified as negative, 8 as positive. The chance that this would happen by chance is equivalent to the chance that we would get at least 45 heads when we flip a fair coin 53 times. For each symptom, both sides of the classification have at least marginally significant *p*-values, i.e., the classifier does well in classifying both those who screen positive and those who screen negative. For eight of the ten symptoms tested, both classifications yielded *p* < 0.001. For all ten symptoms, the total classified correctly (column 6) was significant with *p* < 0.0003. This provides confidence that the neuroelectric measures which comprise the classifier are related to the symptoms. For each symptom, the regions whose measures were included are shown in Table 3 and Table 4. 

### 3.2. CamCAN vs. TEAM-TBI Cohort

MEG recordings and high resolution T1-weighted MR imaging (MRI) were obtained from 613 CamCAN subjects at baseline, 254 at follow-up, 63 TEAM-TBI subjects at baseline and 40 at follow-up. Regional measures of neuroelectric activity for 68 cortical regions and 68 adjacent white matter regions were combined into classifiers using stepwise linear classification [27]. Classification accuracies with *p*-values are shown in Table 5. The regions which contributed to the classifier are listed in Table 6.

### 3.3. Test-Retest Reliability

Regional measures of neuroelectric activity for 68 cortical and 68 adjacent white matter regions were extracted from the resting and task MEG recordings for each member of the CamCAN cohort. These values were used to assess short-term and long-term repeat reliability. For short-term, values from the baseline resting vs. task recordings obtained in the same sitting were used. For long-term, values from the baseline vs. follow-up resting recordings were used (*mean* interval = 16 months). The long-term correlations and *mean* differences for each region are listed in Appendix A Table A2. 

**Figure 3:**
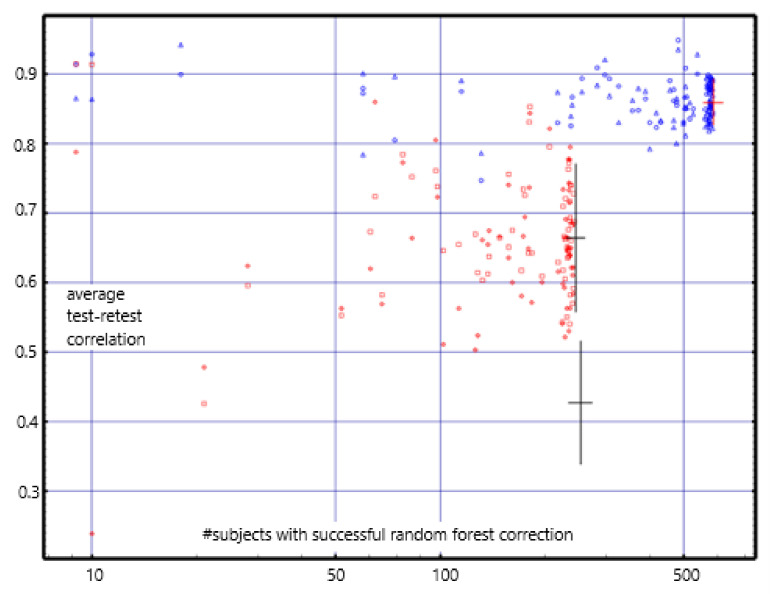
The y-coordinate of each dot is the average test-retest correlation for a region of interest. The x-coordinate is the number of subjects for whom the random forest empty-room adjustment was successfully applied for that ROI. Short-term test-retest reliability is shown in blue, i.e., resting vs. task in the same sitting. Long-term (*mean* 16 months) reliability is shown in red. The error bars show the mean ± 1.0 standard deviation for the short-term values (red bar), long-term (upper black bar), and for the less accurate empty-room correction used in previous work (lower black bar) [10]. The improvement in test-retest reliability with application of the random forest empty-room correction is significant with *p* < 10^−26^, *t* = 13.953, *df* = 126.3 (Welch’s t-statistic). Only the 68 cortical ROI measures were used for the error bar and t-statistic calculations since these are the ROIs to which both random forest and the previous empty-room correction were applied.

The correlations are centered about 0.85 for the short-term; they were centered about 0.8 using the previously reported empty-room correction [12]. For the long-term, they are centered about 0.67; they were centered about 0.45 with the previous correction (see Figure 3). The *mean* long-term differences are consistently near zero and are much less than those found with the previous empty-room correction. Test-retest reliability with values with the random forest empty-room correction is high in both the short-term and long-term and is consistently better than with the previously reported empty-room correction.

Note that the resting recordings were obtained with eyes closed while the task recordings were obtained with eyes open. Yet the comparisons of these, reported here as short-term correlations are quite high, demonstrating that these measures, unlike fMRI measures, are relatively insensitive to eyes open vs. eyes closed. 

### 3.4. Differential Activity: Cortical vs. Adjacent White Matter Regions

For each of 68 cortical regions, freesurfer identifies an adjacent white matter region with maximum thickness of 5 mm. For each such pair of regions the difference in activity can be tested for significance by comparing the observed current counts within the regions to the expected counts given the volumes of the regions. This is not only a test of the spatial resolution of the referee consensus solver, but in addition may provide useful neurophysiological information. See the discussion for additional comments.

For most regions, there are thousands of counts so there is considerable statistical power to identify differences using the χ^2^ statistic. For each of the 613 baseline CamCAN subjects, there are 68 cortex/white matter region pairs, i.e., 41,684 in total. The random forest empty-room correction was successful for 70% of these pairs, i.e., for 29,264, To reduce false positives due to the large number of comparisons, *p* < 10^−8^ was used as the threshold for significance. 

9018 (30.8%) of the pairs demonstrated greater cortical than white matter activity. This supports the claim that the solver’s resolution is less than 5 mm. Surprisingly 15,137 (51.7%) of the pairs demonstrated greater white matter than cortical activity. Additional comments may be found in the discussion.

## 4. Discussion

MEG-derived regional brain measures of tonic neuroelectric activation were tested for long-term test-retest reliability in a large normative cohort, CamCAN, and for sensitivity to symptoms and clinical syndromes in a chronic TBI cohort, TEAM-TBI. The studied symptoms were insomnia, depression, anxiety, and somatization. The clinical syndromes were chronic pain, psychological health, and cognitive, ocular, vestibular, and sleep dysfunction. Good test-retest reliability was found as well as sensitivity to all four symptoms and all six clinical syndromes. Hence the measures reported here may prove of significant clinical utility in diagnosis and treatment. In addition, the measures enable classification of each individual into her/his cohort, i.e., normative vs chronic TBI. Hence the measures may prove useful as biomarkers for TBI.

The analysis and all results were obtained “by region”. Since we are seeking measures with good test-retest reliability and which can be compared between subjects, the volumetric units we use are regions, i.e., volumes which can be reliably and automatically identified because they are common to the anatomically normal human brain. As more detailed atlases with finer structures are developed, the measures reported here will be recomputed and tested for those volumes. For the present, the volumes to which the measures reported here apply are the regions identifiable with freesurfer 5.3 [11,12].

Each regional value which demonstrates long-term test-retest reliability is a measure of regional neuroelectric tonus, i.e., the static level of regional neuroelectric activation. Elevated or reduced regional tonus within an individual may prove emblematic of tonic alterations in network function. The ability to assess many such regional measures simultaneously may provide substantive useful information which is complementary to the measures which have specificity to TBI, e.g., blood born markers [30,31], MEG-derived slow waves [32,33,34]. These patterns of altered regional tonus may prove useful in monitoring response to treatment.

Analysis of the patterns may enable identification of regions to target for treatment. In particular, the localization of the measures to several centimeter^3^ regions is comparable to the localization precision of trans-cranial magnetic stimulation (TMS) [35,36,37,38]. The deviations seen in a particular individual may prove sufficient to identify individualized target regions for TMS, unlike the practice of standardized targeting of left and/or right prefrontal cortex currently in use for major depression [39,40,41,42].

### 4.1. Potential Clinical Utility

This study was undertaken to utilize and assess MEG-derived measures for the diagnosis and monitoring of treatment for chronic sequellae of TBI. We report results which demonstrate (a) sensitivity to the presence/absence of insomnia, somatization, depression, anxiety, chronic pain, psychological health, and sleep, vestibular, oculomotor, and cognitive dysfunction (Table 2) and (b) sensitivity to history of concussion and/or chronic symptoms (Table 5). We cannot directly tie these MEG-results to TBI. However, for clinical purposes, the etiology may not matter so long as we can use the measures to more effectively diagnose and treat.

The symptomatic identification accuracies shown in Table 2 are reliably significant, and the percentages are approaching what is needed for this classification method to be useful clinically. It is likely that classification accuracy can be increased by (a) refining the measures of neuroelectric activity and by (b) using nonlinear or machine-learning classification methods,

The primary results of the study combine the information contained in many regional neuroelectric measures into patterns of brain activity which are related to chronic symptoms in chronic TBI. We also report cohort-wide differences in regional activity (Table 5 and Table 6). These are findings which suggest ways to study the mechanisms which underlie presentation and recovery from symptoms. Productive scientific use of these findings may be complemented by a working theoretical conjecture. To this end, we propose a phantom pain conjecture: all symptoms of psychological distress result from hyper- or hypo-activity in brain regions responsible for attention and response to pain. In support of this conjecture, many regions which show fMRI-derived differential activation in response to painful stimuli ([43], Table 1) show differential activation in MEG-derived measures in the TEAM-TBI cohort when compared with the CamCAN cohort (Table 6), e.g., cuneus, fusiform, paracentral, precuneus, and medialorbitofrontal cortex and the adjacent white matter.

### 4.2. Test-Retest Reliability

We report short-term (1-h, *n* = 613) and long-term (*mean* 16-months, *n* = 253) test-retest reliability for the CamCAN normative cohort for each of 103 brain regions. We use Pearson’s correlation and *mean* difference in test-retest values, Appendix A Table A2 and Figure 3. The difference measure may be used to correct a follow-up measure to compare with a baseline.

Short-term repeat reliability ranged around a *mean* correlation of 0.85. Long-term repeat reliability ranged around a *mean* correlation of 0.67 with *mean* average difference as high as |*z-score*| = 0.147.

A survey of recent test-retest reliability reports shows reliability ranging widely. As would be expected, test-retest reliability is more consistent for time-locked measures in evoked response paradigms [44,45,46,47,48,49,50,51]. For free-running paradigms, measures are consistently resting connectivity confined to frequency bands [52,53,54,55,56,57,58]. The test-retest intervals in most studies are days to weeks. Recasens et al. [47] report results from a 7-week interval, however, both Piitulainen et al. [57] and Dunkley et al. [48] report results for intervals greater than one year. Over all the reports, the maximum number of subjects was 40 [46]. Cohorts with clinical diagnoses were reported by Candelaria-Cook et al. [55] (psychosis) and Dunkley et al. [48] (PTSD).

Both short-term and long-term reliability values we report compare favorably with all others. For the work reported here (a) the *n*’s are much larger, (b) the long-term interval is 16 months, (c) the measures are free-running rather than synchronized to an event, and (d) the measures are from raw rather than averaged data.

### 4.3. Currents Localized to White Matter Regions

We report profuse detectable neuroelectric activity from the white matter with positive differentials in favor of adjacent white matter for 62.7% of those pairs for which the differential is significant with threshold: *p* < 10^−8^. Both previously reported measurements and neurophysiological understanding speak to the plausible validity of these findings. MEG-derived evoked responses from thalamocortical fibers have been reported [59,60]. The source magnetic fields were presumed due to synchronous volleys of action potentials, APs. Each AP produces a travelling current quadrupole. The approximate amplitude has been estimated at 100 Amp^−15^ m in an unmyelinated axon [7] with separation of 1 mm between the two dipoles forming the quadrupole assuming a propagation velocity of 1 m/s. It is presumed that the velocity is greater in the myelinated fibers which comprise the white matter. Hence the velocity and dipole separation would be greater. This would decrease the distance-dependence of the magnetic field strength and so enhance the detectability of this activity. In addition, the magnetic field due to an action potential in a single axon has been directly measured at about 150 × 10^−12^ Tesla [60].

A trivial explanation of the profuse findings we report is that cortical activity is localized in nearby white matter due either to poor resolution or to head movements. The robust finding of differential activity between adjacent cortical and white matter ROIs [10] argues against this. So too does the design of the method which relies on gradients between points within the brain that are 1 mm apart [8,9,61,62], coupled with the use of once per second corrections to the forward solution using continuous head positioning information.

Under the assumption that the white matter is, in fact, the source of profuse measurable neuroelectric activity, the measured magnetic field components can only be due to synchronous volleys of APs. These would produce transient longitudinal intra-axonal currents which are nearly synchronous in many parallel running axons due to near simultaneous passage of propagating APs.

The detected magnetic field waveforms are envelopes which follow the high frequency waveforms of several AP volleys in sequence. The envelope of a single highly synchronized AP volley would require well under 10 ms to rise and fall. Hence this type of activity would be dominated by high frequency content. This is consistent with the observation that the yield of the solver improves when the low pass cut-off with which the signals are preprocessed is increased from 150 Hz to 330 Hz ([8], Figure 3). It is also consistent with the observation from a typical task recording that the frequency content of the current waveforms includes profuse resonant activity with frequency content above 70 Hz [63].

Additional work outside the scope of the present study is needed to understand the mechanisms which underlie the detection of profuse activity localized to the white matter.

### 4.4. CamCAN vs. TEAM-TBI Differences

The robust differences seen between the two cohorts must be interpreted with caution. We cannot rule out the possibility that those differences are due to differences in the scanners or the scanner noise environments. Given the robustness of the differences, this question can be answered by running a cohort of 40 neurologically normal individuals in the scanner used for the TEAM-TBI cohort. The classifiers developed for the present study can be applied to the measures from such a control cohort. If they are different from the CamCAN cohort, then the differences between the CamCAN and TEAM-TBI cohorts must be presumed to be due to differences in the scanners.

A second potential confound to these results is that the CamCAN resting recordings were obtained with eyes closed whereas the Team-TBI recordings were obtained with eyes open. To test this, consider that the short-term test-retest reliability results were obtained by comparing baseline CamCAN resting (eyes closed) with CamCAN sensorimotor task (sitting, eyes open). The test-retest reliability is very good, i.e., the differences between resting and task are very small. In addition, linear classifiers fail to distinguish well between baseline CamCAN resting and task recordings. Hence this difference in recording conditions, i.e., eyes closed vs. eyes open, does not account for the differences between the cohorts.

It is noteworthy that differences found between TEAM-TBI cohort members with and without specific symptoms is not affected by these questions. The same applies to the test-retest reliability results. Only the cause of the differences between the cohorts is in question.

Under the assumption that the differences found between cohorts are due to differences from the norm in the neuroelectric brain activity of those with TBI, correspondences between the normal vs. TBI classification results we report and those reported by others may be useful. The high classification accuracy found between CamCAN and TEAM-TBI cohorts, i.e., greater than 93% (Table 5), provides confidence in the validity of the regions whose measures contribute most to the classifier (Table 6).

At present, we can only speculate what the neurophysiologic mechanisms are which tie altered regional activation, functional connectivity, and symptoms together. The growth in our ability to reliably measure such alterations and to target specific regions with drug and TMS therapies may enable us to understand those mechanisms and to more effectively treat them.

## Figures and Tables

**Table 1:** Coincidence rates at baseline (*n* = 62) *for* pairs of symptoms and adjudicated clinical syndromes. Clinical syndrome names are highlighted in gray. See text for details. Insomnia threshold score = 15 (ISI). Somatization, depression, and anxiety threshold *t-statistic* = 63 (BSI). The six clinical syndromes were assessed by consensus at adjudication.

	Somatization	Depression	Anxiety	Cognitive	Pain	Ocular	Psych Health	Sleep	Vestibular	
insomnia	0.67	0.61	0.71	0.64	0.57	0.52	0.45	0.67	0.58	insomnia
somatization		0.66	0.70	0.61	0.52	0.49	0.54	0.58	0.55	somatization
depression			0.66	0.43	0.52	0.55	0.48	0.48	0.61	depression
anxiety				0.59	0.50	0.51	0.52	0.62	0.55	anxiety
cognitive					0.55	0.42	0.51	0.63	0.42	cognitive
pain						0.53	0.52	0.46	0.53	pain
ocular							0.39	0.43	0.64	ocular
psych health								0.60	0.47	psych health
sleep									0.41	sleep
	somatization	depression	anxiety	cognitive	pain	ocular	psych health	sleep	vestibular	

**Table 2:** **Clinical trajectory classification accuracy using regional activation.** 136 cortical and adjacent white matter regional measures were trained as linear classifiers by group determined by adjudicated clinical trajectory opinions. Jackknifed classification accuracies are shown. Trajectory opinions and regional neuroelectric measures were used from both baseline (*n* = 62) and follow-up (*n* = 40) sessions for all subjects of the TEAM-TBI cohort. The *p-values* were obtained using χ^2^ with *df* = 1 (column 5) and *df* = 2 (column 7). *p*-values > 0.001 are not reported. The trajectories were coded 0/1. For follow-up trajectories, code was set to 0 if the subject had improved (ocular) or fully recovered (all others).

Insomnia	Classified Negative	Classified Positive	Percentage	*p*-Value <	Right/Wrong	*p*-Value <
clinically negative	45	8	84.9%	10^−6^	80/20	10^−7^
clinically positive	12	35	74.5%	10^−6^		
**somatization**	classified negative	classified positive	percentage	*p*-value <	right/wrong	*p*-value <
clinically negative	46	10	82.1%	10^−5^	79/19	10^−7^
clinically positive	9	33	78.6%	0.0003		
**depression**	classified negative	classified positive	percentage	*p*-value <	right/wrong	*p*-value <
clinically negative	52	14	78.8%	10^−5^	78/20	10^−7^
clinically positive	6	26	81.2%	0.0005		
**anxiety**	classified negative	classified positive	percentage	*p*-value <	right/wrong	*p*-value <
clinically negative	39	13	75.0%	0.0004	69/29	0.0003
clinically positive	16	30	65.2%	0.04		
**cognitive**	classified negative	classified positive	percentage	*p*-value <	right/wrong	*p*-value <
clinically negative	33	3	91.7%	10^−6^	91/8	10^−15^
clinically positive	5	58	92.1%	10^−10^		
**headache**	classified negative	classified positive	percentage	*p*-value <	right/wrong	*p*-value <
clinically negative	42	8	84.0%	10^−5^	88/12	10^−12^
clinically positive	4	46	92.0%	10^−8^		
**ocular**	classified negative	classified positive	percentage	*p*-value <	right/wrong	*p*-value <
clinically negative	51	15	77.3%	10^−4^	74,25	10^−5^
clinically positive	10	23	69.7%	0.03		
**psych** health	classified negative	classified positive	percentage	*p*-value <	right/wrong	*p*-value <
clinically negative	17	2	89.5%	0.0006	88/11	10^−13^
clinically positive	9	71	88.8%	10^−11^		
**sleep**	classified negative	classified positive	percentage	*p*-value <	right/wrong	*p*-value <
clinically negative	27	2	93.1%	10^−5^	90/9	10^−14^
clinically positive	7	63	90.0%	10^−10^		
**vestibular**	classified negative	classified positive	percentage	*p*-value <	right/wrong	*p*-value <
clinically negative	48	8	85.7%	10^−7^	83/16	10^−9^
clinically positive	8	35	81.4%	10^−4^		

**Table 3:** The left-side regions whose neuroelectric activity values contributed to the symptom-specific classifiers reported in Table 2 are shown. Activity in regions marked “↑” was higher in those who screened positive; those marked “↓“ were lower.

Left	Cortex										Adjacent White Matter									
	Insomnia	Somatization	Depression	Anxiety	Cognitive	Chronic Pain	Ocular	Psych Health	Sleep	Vestibular	Insomnia	Somatization	Depression	Anxiety	Cognitive	Chronic Pain	Ocular	Psych Health	Sleep	Vestibular
bankssts		↓						↓		**1**										**69**
caudalanteriorcingulate		↑								↓					↓					**70**
caudalmiddlefrontal										**3**										**71**
cuneus					↓					**4**									↓	**72**
entorhinal							↑			**5**	↓								↓	**73**
frontalpole										**6**										**74**
fusiform										**7**					↑					**75**
inferiorparietal			↑		↓		↑			**8**						↑				**76**
inferiortemporal										**9**								↓	↑	**77**
insula	↑									**10**			↑					↑		**↓**
isthmuscingulate										**11**										**79**
lateraloccipital										**12**										↓
lateralorbitofrontal			↓							**13**									↓	**81**
lingual										**14**						↓				**82**
medialorbitofrontal										**15**										**83**
middletemporal		↓								**16**										**84**
paracentral										**17**										**85**
parahippocampal										**18**		↑								**86**
parsopercularis						↓			↑	**19**			↓						↓	**87**
parsorbitalis										**20**										**88**
parstriangularis						↑				**21**										**89**
pericalcarine										**22**										**90**
postcentral										**23**										**91**
posteriorcingulate				↓						**24**										**92**
precentral										**25**					↑					**93**
precuneus										**26**										**94**
rostralanteriorcingulate					↑					**27**			↓		↑					↓
rostralmiddlefrontal										**28**										**96**
superiorfrontal		↑								**29**								↓		**97**
superiorparietal					↑			↓	↑	↓										**98**
superiortemporal						↑		↑		**31**									↓	**99**
supramarginal										**32**										**100**
temporalpole						↑				↑										**101**
transversetemporal					↓					**34**			↑							**102**

**Table 4:** The right-side regions whose neuroelectric activity values contributed to the symptom-specific classifiers reported in Table 2 are shown. Activity in regions marked “↑” was higher in those who screened positive; those marked “↓“ were lower.

	Cortex										Adjacent White Matter									
	Insomnia	Somatization	Depression	Anxiety	Cognitive	Chronic Pain	Ocular	Psych Health	Sleep	Vestibular	Insomnia	Somatization	Depression	Anxiety	Cognitive	Chronic Pain	Ocular	Psych Health	Sleep	Vestibular
bankssts										**35**	↑									**103**
caudalanteriorcingulate									↓	**36**										**104**
caudalmiddlefrontal						↑				↓										↑
cuneus										**38**										↓
entorhinal										**39**										**107**
frontalpole										**40**										**108**
fusiform										**41**					↑					**109**
inferiorparietal										**42**										**110**
inferiortemporal										↓										**111**
insula										**44**										**112**
isthmuscingulate							↑			**45**										**113**
lateraloccipital										**46**										**114**
lateralorbitofrontal				↑						**47**										↑
lingual				↓				↓		**48**										**116**
medialorbitofrontal		↓	↓	↓				↓		↓		↑			↑					**117**
middletemporal										**50**	↓				↓					**118**
paracentral						↑				**51**										**119**
parahippocampal										**52**		↓								**120**
parsopercularis									↑	**53**	↓									**121**
parsorbitalis										**54**							↑			**122**
parstriangularis					↓					**55**	↑							↓		**123**
pericalcarine									↓	↓										**124**
postcentral				↓					↓	**57**										**125**
posteriorcingulate						↑				**58**	↓					↓				**126**
precentral				↑						**59**			↓			↑	↑			**127**
precuneus						↑				**60**						↓				**128**
rostralanteriorcingulate						↑				**61**			↑							**129**
rostralmiddlefrontal								↑		**62**						↓				**130**
superiorfrontal										**63**										**131**
superiorparietal		↓								**64**										**132**
superiortemporal										**65**										**133**
supramarginal	↓	↓			↓					↓										**134**
temporalpole									↓	**67**										**135**
transversetemporal					↑	↓				**68**										**136**

**Table 5:** 68 cortical and 68 adjacent white matter regional measures were trained as linear classifiers by cohort using the baseline CamCAN and TEAM-TBI measures. Jackknifed classification accuracies are shown. The results highlighted in light gray were obtained when the regions which were selected for the first classification analysis were excluded. The results highlighted in darker gray were obtained when the regions which were selected for the both the first and second classification analyses were excluded. See the text for details.

1st Step	Classified as CamCAN	Classifiedd asTEAM-TBI	Percentage	*p*-Value
CamCAN baseline	602	11	98.2%	10^−125^
CamCAN follow-up	236	9	96.3%	10^−46^
TEAM-TBI baseline	4	59	93.7%	10^−11^
TEAM-TBI follow-up	8	32	80.0%	10^−3^
**2nd step**	CamCAN	TEAM-TBI	percentage	*p*-value
CamCAN baseline	578	35	94.3%	10^−105^
CamCAN follow-up	231	14	94.3%	10^−42^
TEAM-TBI baseline	3	60	95.2%	10^−12^
TEAM-TBI follow-up	3	37	92.5%	10^−7^
**3rd step**	CamCAN	TEAM-TBI	percentage	*p*-value
CamCAN baseline	534	79	87.1%	10^−74^
CamCAN follow-up	221	24	90.2%	10^−35^
TEAM-TBI baseline	6	57	90.5%	10^−9^
TEAM-TBI follow-up	4	36	90.0%	10^−6^

**Table 6:** The regions whose neuroelectric activity values contributed to the cohort-specific classifiers reported in Table 5 are shown. Activity in regions marked ↑ was higher in the Team-TBI cohort; those marked ↓ were lower. Regions marked ↑↑ or ↓↓ made the largest statistical contribution to the classifier. The results for the 2nd step are highlighted in light gray and for the 3rd step are highlighted in darker gray as in Table 5. These were obtained when the regions which were selected for the 1st (light gray) and 1st and 2nd (dark gray) classification analyses were excluded.

Left			Right	
Cortex	White Matter		Cortex	White Matter
		bankssts		↑
↓	↓↓	caudalanteriorcingulate	↓	↓↓
		caudalmiddlefrontal		
	↑	cuneus	↑↑	↑
↓	↓	entorhinal		
		frontalpole		
↓↓	↓	fusiform	↓	↓
	↓	inferiorparietal		↓
	↓	inferiortemporal		
↑↑	↑	insula	↑	
	↑↑	isthmuscingulate	↑	↑↑
		lateraloccipital		
		lateralorbitofrontal		
↓	↑	lingual	↓	
	↑	medialorbitofrontal	↓	
		middletemporal	↑	↑
↑	↑	paracentral		
	↑↑	parahippocampal		↑
	↑	parsopercularis		
	↑↑	parsorbitalis		↑↑
↓	↑	parstriangularis	↓	↑
		pericalcarine	↑	
		postcentral		
↑	↓	posteriorcingulate		↓
↑		precentral		
↓↓	↓	precuneus	↓↓	↓↓
	↑	rostralanteriorcingulate	↓	↑↑
↓		rostralmiddlefrontal	↓	
		superiorfrontal		
		superiorparietal		↑
		superiortemporal		↑
	↓↓	supramarginal		↓
↓	↑	temporalpole		
↑↑	↑	transversetemporal	↑	

## Data Availability

The CamCAN datasets are available on request from the Cambridge Centre for Ageing and Neuroscience (CamCAN) at the University of Cambridge, UK, https://www.cam-can.org/, most recently accessed on 1 December 2021. The TEAM-TBI datasets are available on request from the Federal Interagency Traumatic Brain Injury Research (FITBIR) Informatics System at the National Institutes of Health, USA, https://fitbir.nih.gov/, most recently accessed on 1 December 2021. The collection of currents identified by the referee consensus solver (≈5 TBytes) are available on request from the corresponding author, kriegerd@upmc.edu.

## Appendix A

**Table A1:** Atlas for measures with random forest empty-room correction. Regional measures of neuroelectric activity for 68 cortical and 68 adjacent white matter regions were extracted from both the baseline (*n* = 613) CamCAN recordings. The columns labelled “*mean*” and “*s.d.*” contain the *means* and *standard deviations* for the baseline current density measures These density measures were transformed to *z-scores*. The columns labelled “*n*” show the number of subjects for whom the random forest classification yielded significant accuracy for resting vs. empty-room recordings. Only those subjects’ measures were used to compute the densities listed in the atlas.

Left							Right					
Cortex			Adjacent White Matter				Cortex			Adjacent White Matter		
*n*	*mean*	*s.d*	*n*	*mean*	*s.d*		*n*	*mean*	*s.d.*	*n*	*mean*	*s.d.*
530	1.256	0.345	553	1.206	0.337	bankssts	515	1.230	0.368	532	1.177	0.321
155	0.825	0.353	416	0.758	0.349	caudalanteriorcingulate	173	0.735	0.360	384	0.701	0.353
562	0.948	0.353	573	0.983	0.400	caudalmiddlefrontal	543	0.911	0.369	547	0.938	0.409
360	0.889	0.291	389	0.969	0.325	cuneus	368	0.864	0.266	378	0.932	0.303
517	1.291	0.333	149	1.431	0.386	entorhinal	476	1.304	0.355	125	1.537	0.419
0	0	0	0	0	0	frontalpole	6	1.479	0.556	0	0	0
608	1.020	0.201	609	1.008	0.221	fusiform	607	1.067	0.213	603	1.038	0.243
608	0.992	0.236	602	0.894	0.237	inferiorparietal	606	0.939	0.217	606	0.865	0.212
611	1.230	0.213	608	1.308	0.242	inferiortemporal	610	1.236	0.220	609	1.303	0.256
605	0.989	0.234	608	1.066	0.250	insula	603	1.023	0.248	604	1.121	0.278
208	0.849	0.340	426	0.836	0.331	isthmuscingulate	171	0.859	0.303	408	0.880	0.330
611	1.196	0.218	608	1.225	0.236	lateraloccipital	610	1.175	0.226	608	1.228	0.268
601	1.124	0.251	596	1.184	0.313	lateralorbitofrontal	601	1.149	0.267	596	1.186	0.324
599	0.871	0.230	600	0.970	0.256	lingual	599	0.863	0.224	594	0.941	0.264
539	0.853	0.248	511	0.937	0.269	medialorbitofrontal	486	0.800	0.253	420	0.882	0.268
610	1.179	0.225	601	1.346	0.279	middletemporal	611	1.174	0.210	607	1.341	0.267
308	0.574	0.274	420	0.677	0.325	paracentral	324	0.552	0.262	455	0.649	0.301
542	1.256	0.314	467	1.355	0.337	parahippocampal	537	1.273	0.337	469	1.357	0.346
579	1.102	0.309	539	1.055	0.347	parsopercularis	554	1.073	0.341	522	1.008	0.354
325	1.128	0.345	54	1.358	0.421	parsorbitalis	381	1.012	0.362	98	1.340	0.433
532	1.087	0.321	520	1.068	0.338	parstriangularis	556	1.036	0.322	526	1.075	0.345
357	1.016	0.330	543	1.031	0.314	pericalcarine	384	0.970	0.295	538	0.990	0.304
603	0.914	0.235	594	0.965	0.269	postcentral	597	0.884	0.223	594	0.928	0.248
322	0.712	0.322	484	0.724	0.311	posteriorcingulate	305	0.668	0.292	466	0.67	0.281
605	0.944	0.245	609	0.935	0.265	precentral	606	0.914	0.245	605	0.909	0.266
526	0.530	0.235	577	0.663	0.249	precuneus	528	0.529	0.230	580	0.680	0.246
319	0.896	0.319	409	0.917	0.331	rostralanteriorcingulate	184	0.951	0.318	246	0.939	0.321
603	0.823	0.258	603	0.933	0.296	rostralmiddlefrontal	606	0.785	0.278	598	0.906	0.308
599	0.674	0.230	595	0.774	0.292	superiorfrontal	603	0.651	0.237	598	0.741	0.284
606	0.879	0.246	607	0.929	0.255	superiorparietal	604	0.854	0.265	601	0.906	0.280
611	1.164	0.191	610	1.227	0.223	superiortemporal	610	1.160	0.207	607	1.248	0.247
604	0.933	0.244	593	0.947	0.275	supramarginal	599	0.903	0.258	595	0.942	0.268
514	1.171	0.343	55	1.545	0.335	temporalpole	533	1.297	0.325	47	1.553	0.420
223	1.269	0.429	71	1.377	0.432	transversetemporal	109	1.316	0.379	18	1.441	0.378

**Table A2:** Test-retest reliability for measures with random forest empty-room correction. *n* = 245. The regional measures of neuroelectric activity for 68 cortical and 68 adjacent white matter regions were extracted from both the baseline (*n* = 613) and 16-month follow-up (*n* = 245) CamCAN recordings. The columns labelled “corr” and “*diff.*” contain the *correlation* and *difference* between the baseline and follow-up measures. The columns labelled “*n*” show the number of subjects for whom the random forest classification yielded significant accuracy for resting vs. empty-room recordings for both baseline and follow-up.

Left							Right					
Cortex			Adjacent White Matter				Cortex			Adjacent White Matter		
*n*	*corr*	*diff*	*n*	*corr*	*diff*		*n*	*corr*	*diff*	*n*	*corr*	*diff*
148	0.664	0.037	175	0.725	0.095	bankssts	148	0.666	−0.024	175	0.694	−0.138
9	0.914	−0.007	83	0.752	−0.002	caudalanteriorcingulate	9	0.787	−0.058	83	0.663	0.020
179	0.642	−0.089	183	0.642	−0.035	caudalmiddlefrontal	179	0.648	0.004	183	0.571	0.069
97	0.760	0.096	98	0.738	0.070	cuneus	97	0.804	0.042	98	0.722	0.007
137	0.612	−0.093	7	0.062	−0.028	entorhinal	137	0.654	−0.072	7	0.562	−0.024
0	0	0	0	0	0	frontalpole	0	0	0	0	0	0
238	0.739	0.096	233	0.763	0.060	fusiform	238	0.686	0.017	233	0.777	−0.040
235	0.688	0.063	234	0.772	−0.024	inferiorparietal	235	0.714	0.022	234	0.743	−0.007
241	0.591	0.117	237	0.660	0.107	inferiortemporal	241	0.583	−0.004	237	0.649	0.010
235	0.717	0.076	236	0.740	0.037	insula	235	0.777	0.097	236	0.794	0.051
28	0.595	0.041	126	0.669	0.015	isthmuscingulate	28	0.623	0.062	126	0.503	0.039
241	0.728	0.091	236	0.693	0.085	lateraloccipital	241	0.621	0.072	236	0.563	0.034
235	0.540	0.088	225	0.617	0.044	lateralorbitofrontal	235	0.639	0.015	225	0.598	−0.023
229	0.720	0.080	225	0.709	0.073	lingual	229	0.691	0.000	225	0.733	0.024
138	0.637	0.025	113	0.654	−0.006	medialorbitofrontal	138	0.674	0.006	113	0.562	−0.059
242	0.687	0.032	231	0.650	−0.001	middletemporal	242	0.683	−0.005	231	0.646	0.017
63	0.673	0.062	102	0.645	0.058	paracentral	63	0.619	0.031	102	0.510	0.065
171	0.617	−0.097	128	0.614	−0.050	parahippocampal	171	0.580	−0.105	128	0.523	−0.099
196	0.609	−0.088	161	0.675	−0.058	parsopercularis	196	0.600	0.017	161	0.599	0.079
65	0.723	−0.077	3	−0.370	−0.008	parsorbitalis	65	0.859	0.015	3	0.561	−0.005
173	0.733	0.016	157	0.650	0.036	parstriangularis	173	0.666	0.085	157	0.635	0.048
78	0.784	0.000	180	0.830	−0.034	pericalcarine	78	0.772	−0.057	180	0.736	−0.024
231	0.550	−0.070	224	0.542	−0.072	postcentral	231	0.562	−0.026	224	0.540	0.029
68	0.581	0.056	132	0.603	−0.042	posteriorcingulate	68	0.568	−0.037	132	0.660	−0.032
236	0.582	−0.097	234	0.637	−0.031	precentral	236	0.638	−0.013	234	0.530	0.016
181	0.852	−0.073	206	0.795	−0.058	precuneus	181	0.843	−0.051	206	0.821	0.002
21	0.425	−0.017	52	0.552	−0.036	rostralanteriorcingulate	21	0.478	−0.034	52	0.562	0.009
233	0.675	−0.030	228	0.604	−0.016	rostralmiddlefrontal	233	0.649	0.012	228	0.521	0.062
233	0.645	0.012	228	0.661	0.022	superiorfrontal	233	0.651	−0.052	228	0.664	−0.002
232	0.635	−0.015	234	0.646	−0.012	superiorparietal	232	0.664	0.053	234	0.732	0.015
240	0.569	0.100	238	0.682	0.085	superiortemporal	240	0.610	0.030	238	0.621	−0.029
227	0.666	−0.001	218	0.629	−0.039	supramarginal	227	0.592	−0.128	218	0.615	−0.147
157	0.756	0.023	0	0	0	temporalpole	157	0.740	0.001	0	0	0
10	0.913	0.029	0	0	0	transversetemporal	10	0.238	−0.002	0	0	0
